# Cell surface and cell outline imaging in plant tissues using the backscattered electron detector in a variable pressure scanning electron microscope

**DOI:** 10.1186/1746-4811-9-40

**Published:** 2013-10-17

**Authors:** Mark J Talbot, Rosemary G White

**Affiliations:** 1 Division of Plant Industry, Commonwealth Scientific and Industrial Research Organisation, Canberra ACT 2601, Australia

**Keywords:** Backscattered-electron imaging, Plant cell outlines, Image analysis, *Arabidopsis thaliana*

## Abstract

**Background:**

Scanning electron microscopy (SEM) has been used for high-resolution imaging of plant cell surfaces for many decades. Most SEM imaging employs the secondary electron detector under high vacuum to provide pseudo-3D images of plant organs and especially of surface structures such as trichomes and stomatal guard cells; these samples generally have to be metal-coated to avoid charging artefacts. Variable pressure-SEM allows examination of uncoated tissues, and provides a flexible range of options for imaging, either with a secondary electron detector or backscattered electron detector. In one application, we used the backscattered electron detector under low vacuum conditions to collect images of uncoated barley leaf tissue followed by simple quantification of cell areas.

**Results:**

Here, we outline methods for backscattered electron imaging of a variety of plant tissues with particular focus on collecting images for quantification of cell size and shape. We demonstrate the advantages of this technique over other methods to obtain high contrast cell outlines, and define a set of parameters for imaging *Arabidopsis thaliana* leaf epidermal cells together with a simple image analysis protocol. We also show how to vary parameters such as accelerating voltage and chamber pressure to optimise imaging in a range of other plant tissues.

**Conclusions:**

Backscattered electron imaging of uncoated plant tissue allows acquisition of images showing details of plant morphology together with images of high contrast cell outlines suitable for semi-automated image analysis. The method is easily adaptable to many types of tissue and suitable for any laboratory with standard SEM preparation equipment and a variable-pressure-SEM or tabletop SEM.

## Background

The analysis of developmental changes in plant cells, tissues and organs often requires quantification of subtle alterations in cell morphology. Measurements of cell size and shape require contrast enhancement of cell boundaries (cell walls or plasma membrane) to produce high-contrast images suitable for subsequent analysis. Methods to enhance plant cell outlines vary in complexity from straightforward imaging of cell wall autofluorescence to lengthy, multistep processing for three-dimensional analysis of tissue architecture by confocal laser scanning microscopy (CLSM) e.g., [1-3]. These methods are often suited only to particular plants, tissues or cell types due to inherent differences in cell or tissue properties across different species.

Some simple methods, such as differential interference contrast of cleared tissue e.g., [4], produce relatively low-contrast images unsuitable for automated image analysis. Cell outline contrast can be increased by staining fresh tissue with, for example, propidium iodide e.g., [5-7], or membrane-binding FM dyes e.g., [8,9] but these stains do not easily penetrate all tissues without pre-treatment, particularly aerial parts of the plant, which are often coated with a waxy cuticle. CLSM can also be used to detect green fluorescent protein (GFP) targeted to the cell surface e.g., [10], but it is not possible to obtain GFP transformants for every plant or tissue under study. In addition, there is often a requirement with CLSM to acquire a 3-dimensional image series to obtain a complete view of tissues with complex shapes. Considerable subsequent computation is then required to extract information about a single layer of cells such as the epidermis from these stacks [5,11,12].

The Scanning Electron Microscope (SEM) has seldom been used to generate images for the purposes of analysis, largely because conventional imaging of biological tissue under high vacuum SEM requires coating the tissue with a conductive metal, which obscures information in the sample irrespective of the tissue and different beam energies [13]. The images obtained provide useful information about overall tissue morphology and surface details, but most analysis packages struggle to correctly discriminate cell outlines using the subtle differences in grey levels in these pseudo-3D images. SEM imaging usually involves detection of secondary electrons (SE), which are sample-derived electrons generated from interaction of the primary electron beam with the top 1–10 nm of the sample surface [13,14]. In contrast, backscattered electrons (BSEs) are beam electrons which have been scattered deeper within the sample. BSEs can provide atomic number contrast in which differences in signal intensity are related to local differences in the average atomic number [14].

In an environmental pressure SEM (EP-SEM) or variable-pressure SEM (VP-SEM), the specimen chamber operates at much lower vacuum due to the presence of an 'imaging gas’ (typically nitrogen). The gaseous environment around the sample helps to reduce charging artefacts at the sample surface [13], and the specimen can be viewed uncoated or in the case of EP-SEM where water is the imaging gas, viewed hydrated with no processing. In a VP-SEM, SE and BSE signals provide a flexible range of options to image biological tissues [15,16] and can reveal detail not previously visible in coated tissue under high vacuum.

Previously we used a BSE detector with VP-SEM to produce images of high contrast cell outlines in uncoated, critical point dried barley leaves for image analysis [17]. In this paper we extend this technique to a wider range of plant tissues, describe how to optimise this protocol and apply it to quantify cell size in leaves of the model plant *Arabidopsis thaliana*. The advantages of this protocol are that it is simple and quick, it enables recording of surface details together with high contrast images for quantitative analysis using freely available software, and is suitable for any laboratory with standard SEM preparation equipment and any VP-SEM, including tabletop models.

## Results

### General structure

**Figure 1:**
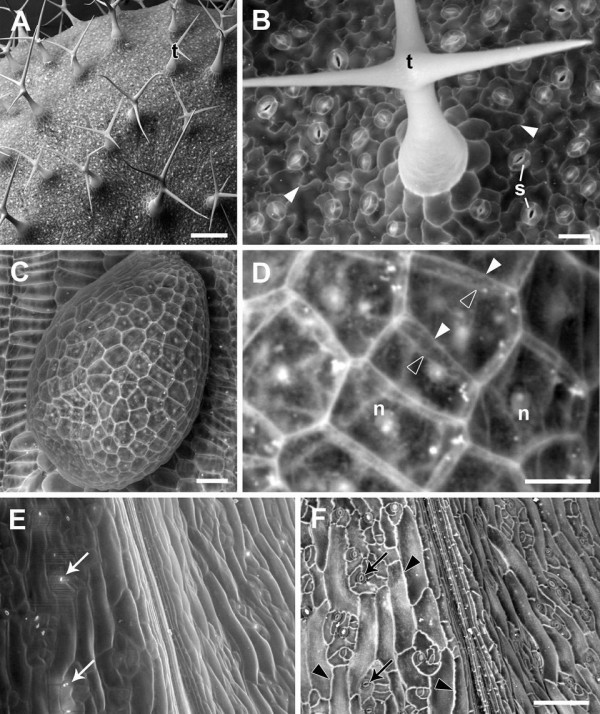
**Critical point dried, uncoated *A. thaliana *tissues examined in the VP-SEM. (A)** Mature rosette leaf showing overall leaf morphology and distribution of trichomes (t). **(B)** Higher magnification view of leaf showing trichome (t), bright cell wall outlines of pavement epidermal cells (arrowheads), and stomatal guard cells (s). **(C)** Developing seed (approx 7 days after flowering), and **(D)** higher magnification view showing 3D views of epidermal cell walls revealed by BSE imaging. Solid arrowheads = junction between epidermal outer periclinal and anticlinal cell walls, open arrowheads = junction between epidermal anticlinal walls and periclinal walls of epidermal and sub-epidermal cells, n = nucleus. Simultaneous capture and comparison of SE **(E)** and BSE **(F)** images of silique outer epidermis. White arrows show stomata charging in (E), black arrows in (F) show same stomata in the BSE image, arrowheads show cell outlines. Accelerating voltage 20 kV and chamber pressure 10 Pa **(A**-**D)** or 80 Pa **(E**, **F)**. Scale bars = 20 μm **(B**, **D)**, 50 μm **(C)**, 100 μm **(E**, **F)** and 200 μm **(A)**.

After solvent fixation and critical point drying, uncoated samples observed using the BSE detector showed good preservation, with tissue topography visible in high contrast on the surface of an *A. thaliana* leaf (Figure 1A) and developing seed (Figure 1C). At higher magnification, the bright signals from leaf epidermal cell walls, trichomes and stomatal guard cells were clear (Figure 1B). In the epidermis of a developing seed both internal and external junctions of anticlinal walls could be seen, revealing the three-dimensional, box-like cell shapes (Figure 1D). In these cells the internal organelles including the nucleus were also visible. The difference between SE and BSE imaging was demonstrated when a section of silique epidermis was viewed simultaneously with the VP-SE detector (Figure 1E) and BSE detector (Figure 1F) at 80 Pa chamber pressure. The SE image revealed surface topography, but some charging of stomatal cells was seen, even at the relatively high chamber pressure used (Figure 1E). Interference from tissue charging was absent in the BSE image, and although there was less topographical detail, bright cell wall outlines were clear (Figure 1F).

### Optimising cell wall outlines in *A. thaliana* leaves with the BSE detector

To extend BSE imaging further, we optimised parameters for producing high contrast images of cell outlines suitable for analysis of cell size and shape. We focused on *A. thaliana*, a model species for studying dicotyledon plant growth and development, but we also included common dicotyledon (cotton) and monocotyledon (barley, wheat, rice and *Brachypodium*) species for comparison. Leaves are ideal for this type of analysis as they are relatively flat, and epidermal cells generally contain little cytoplasm and few chloroplasts, components which add to the BSE signal and complicate image analysis.

#### Accelerating voltage

**Figure 2:**
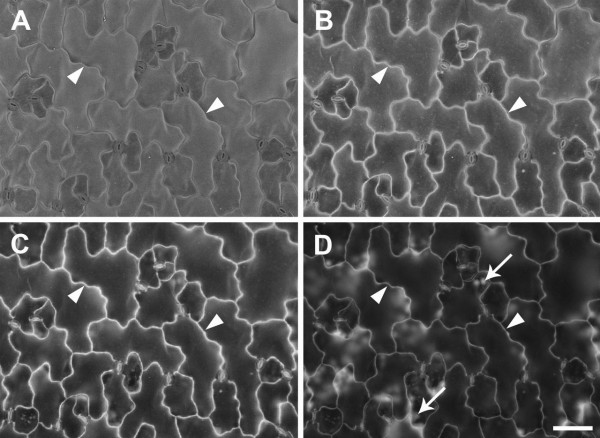
**Effect of accelerating voltage on BSE imaging of cell wall outlines in critical point dried *A. thaliana *rosette leaf.** The same area of a leaf was imaged at accelerating voltages 10 **(A)**, 15 **(B)**, 20 **(C)** or 30 kV **(D)**. Arrowheads indicate cell wall boundaries, arrows indicate chloroplasts in mesophyll cells. Chamber pressure 10 Pa. Scale bar = 60 μm (A–D, bar shown in D).

Varying the accelerating voltage significantly affected the visibility of cell wall outlines. At 10 kV, the surface of *A. thaliana* leaf pavement epidermal cells could be seen but cell outlines were of poor contrast (Figure 2A) and accelerating voltages lower than 10 kV produced noisy BSE images (not shown). Increasing the accelerating voltage to 15 kV substantially increased cell wall contrast, although some signal from the cell surface was still discernible (Figure 2B). Surface details became less obvious at 20 kV, while cell outlines were very prominent (Figure 2C). However, at 30 kV, the beam penetrated further into the tissue, generating signal from the underlying cells and reducing the contribution from anticlinal cell walls (Figure 2D). Based on these results an accelerating voltage of 20 kV was chosen for subsequent imaging of cell outlines in *A. thaliana*.

#### Spot size, working distance, and chamber pressure

**Figure 3:**
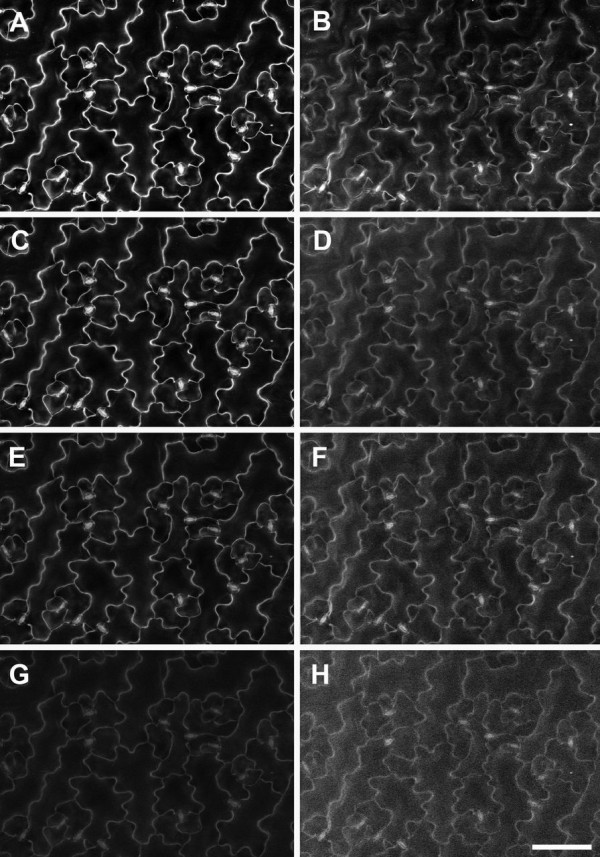
**Effect of chamber gas pressure on imaging of cell wall outlines in critical point dried *A. thaliana *rosette leaf.** BSE **(A**,**C**,**E**,**G)** and SE **(B**,**D**,**F**,**H)** images were collected from the same area of a single leaf, at 10 **(A**, **B)**, 50 **(C**, **D)**, 100 **(E**, **F)**, and 200 Pa **(G**, **H)** chamber pressure, respectively. Brightness and contrast levels were not changed for BSE images. Accelerating voltage 20 kV. Scale bar = 60 μm (A–H, bar shown in H).

Other parameters were optimised to increase the BSE signal from cell outlines. Relatively low magnifications were used to obtain images (100-400×), and a sufficiently large spot size (707 pA probe current) was chosen to increase the signal-noise ratio (SNR). An optimal working distance (WD) of approx. 7 mm yielded maximum signal at 20 kV (Additional file 1). Chamber pressure can be varied across a wide range in a VP-SEM, primarily to reduce charging, although higher chamber pressures lead to beam 'skirting’, scattering of primary beam electrons by the imaging gas, which decreases the SNR [13]. For *A. thaliana* leaves, a pressure range of 10–50 Pa proved optimal (Figure 3A-D) since above 50 Pa, increased noise from electron beam scattering reduced cell wall contrast in both BSE and SE images (Figure 3E-H). A chamber pressure of 10 Pa was used routinely since this was the minimum available and resulted in the brightest and clearest BSE images (Figure 3A). Interestingly, topographical contrast was low with the SE detector at 20 kV, and cell outlines were revealed under these conditions (e.g., Figure 3B,D,F,H). However, cell outline contrast was low compared to BSE images, and in other tissues could not be resolved in SE images (e.g., Figure 1E).

#### Charge reduction

**Figure 4:**
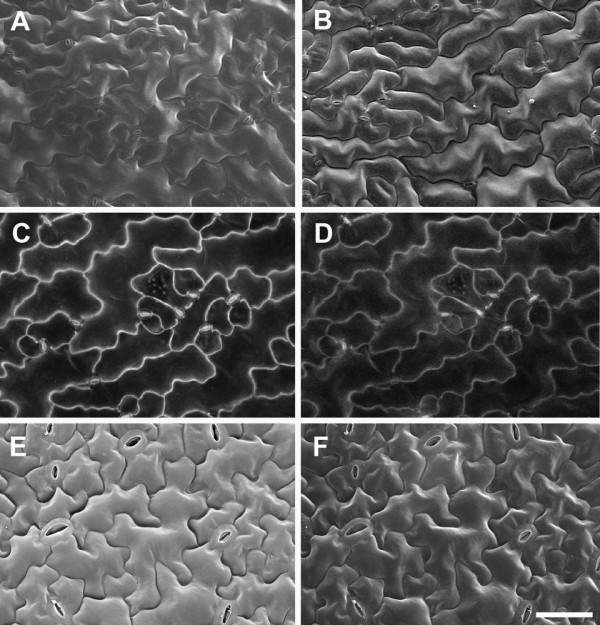
**Reducing charging for imaging surface details and cell wall outlines in critical point dried *A. thaliana *rosette leaves under VP mode.** Uncoated **(A)** and carbon-coated **(B)** leaf imaged at 10 kV accelerating voltage with the VP-SE detector for surface topography. **(C**-**F)** BSE **(C**, **E)** and SE **(D**, **F)** images were collected at 20 kV from the same area of leaf, either coated with carbon **(C**, **D)**, or gold **(E**, **F)**, respectively. Chamber pressure 10 Pa. Scale bar = 60 μm (**A**–**F**, bar shown in **F**).

In some cases imaging of both surface details and high contrast cell wall outlines may be required from the same tissue. We found that the best combination of parameters for BSE imaging of cell wall outlines was 20 kV accelerating voltage and 10 Pa chamber pressure (Figure 2C,3A). However, while the BSE detector is relatively insensitive to charging artefacts, under these conditions topographical contrast was low with the VP-SE detector and charging had a significant impact on SE image quality (e.g., Figure 3B). Therefore it was difficult to simultaneously capture surface topography and cell wall outlines from uncoated tissue with the BSE and VP-SE detectors. Imaging at 10 kV (10 Pa chamber pressure) ameliorated charging with the VP-SE detector and provided reasonable topographic contrast (Figure 4A), which was improved with carbon coating (Figure 4B). Charging was also reduced at 20 kV in carbon-coated tissue, but topographic contrast was not improved at this higher accelerating voltage (Figure 4D). Carbon coating did not affect imaging of cell outlines with the BSE detector (Figure 4C). Therefore, to improve the flexibility of imaging options, the accelerating voltage can be adjusted to capture both surface detail (10 kV, VP-SE or BSE detectors) and cell wall outlines (20 kV, BSE detector) from either uncoated or coated tissue.

**Figure 5:**
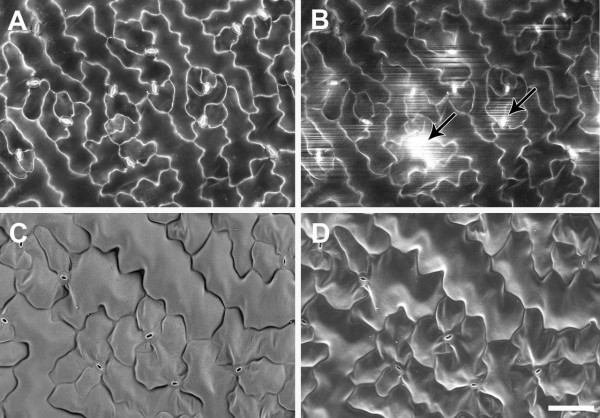
**Effects of carbon or gold-coating on imaging of cell wall outlines in critical point dried *A. thaliana *rosette leaves under high vacuum.** BSE **(A**, **C)** and SE **(B**, **D)** images were collected from the same leaf areas coated with carbon **(A**, **B)** or gold **(C**, **D)**, respectively. Arrows indicate charging artefacts in SE image of carbon-coated leaf **(B)**. Scale bar = 40 μm (**A**–**D**, bar shown in D).

For imaging *A. thaliana* leaves, chamber pressure was kept to a minimum (10 Pa) to maximise SNR (see Figure 3). However, not all tissues image in the same way, and we recommend testing uncoated tissue with both the VP-SE and BSE detectors at different accelerating voltages and chamber pressures to determine the best parameters for imaging, then carbon coat tissue if necessary. If charging remains an issue, contact between the tissue and the carbon tab can be improved by filling gaps between the edges of the tissue and the stub or carbon tab with carbon paste. Images can also be acquired by frame averaging at a faster scan rate to reduce beam dwell time on the sample, rather than line averaging, in which the beam spends longer at one point on the sample causing increased charge build-up. Sputter coating with gold greatly reduces charging but also eliminates the high contrast cell outlines altogether (Figure 4E,F) When observed under conventional high vacuum at 20 kV accelerating voltage, high contrast cell outlines were similarly obtained using the BSE detector (Figure 5A), but there was significant charging with the SE signal (Figure 5B) which could not be avoided by frame averaging. As with low vacuum imaging (Figure 4E, F), gold coating prevented beam penetration below the surface and produced conventional topographical images (Figure 5C,D).

#### Low temperature VP-SEM and extended-pressure-SEM (EP-SEM)

**Figure 6:**
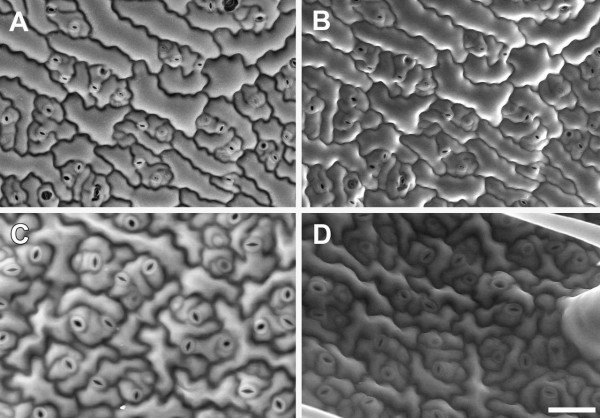
**BSE and SE imaging of hydrated *A. thaliana *rosette leaves.** The same area of leaf was imaged frozen on a Peltier-cooled stage with the BSE **(A)** or SE **(B)** detectors in VP mode (Accelerating voltage 20 kV and chamber pressure 10 Pa). Different leaves were imaged in extended pressure mode with water vapour as the imaging gas with the BSE **(C)** and SE **(D)** detectors. EP-SEM conditions were 82% humidity, 600–700 Pa chamber pressure, 2-3°C Peltier stage temperature, and 20–25 kV accelerating voltage. Scale bar = 40 μm (**A**–**D**, bar shown in D).

A well-known artefact of preparing tissue for SEM is shrinkage during fixation, dehydration and critical point drying [18,19]. This can be improved by concomitant fixation and dehydration in methanol rather than other standard fixatives [20], but we examined whether BSE outlines could be observed in uncoated hydrated tissue, which avoids all processing steps prior to imaging. However, bright cell outlines typical of critical point-dried tissue were not visible in frozen tissue mounted on the Peltier-cooled stage (Figure 6A,B), or when fresh, cooled tissue was observed in the presence of water vapour using the extended pressure capability of the SEM (Figure 6C,D). Interestingly, BSE images of either frozen (Figure 6A) or fresh (Figure 6C) leaves showed dark cell wall outlines due to topographic contrast at cell junctions, which was more evident in frozen material (Figure 6A), and such images could be used for image analysis. However, there are several disadvantages with imaging frozen tissue; (1) without a dedicated liquid nitrogen-temperature cryo-stage attached to the microscope, imaging needs to be completed within 20 minutes of pumping the chamber to pressure, as the tissue will freeze-dry after this time and become distorted; (2) only one leaf can be imaged at a time, because of space available on the stub, and to maximise the number of images taken per leaf before freeze-drying occurs; (3) the tissue cannot be stored after imaging; and (4) there is more cell wall outline contrast in images of critical point dried leaves. Furthermore, extended pressure imaging is inherently difficult, since many variables need to be delicately balanced to retain liquid water in the tissue while minimizing beam damage [16]. In addition, only a limited field of view and range of magnification is available with this method, due to the necessary installation of additional apertures in the beam path and small working distances needed to reduce beam skirting. As with low temperature VP-SEM, the tissue can only be viewed once under EP-SEM.

### BSE imaging of other plant tissues

**Figure 7:**
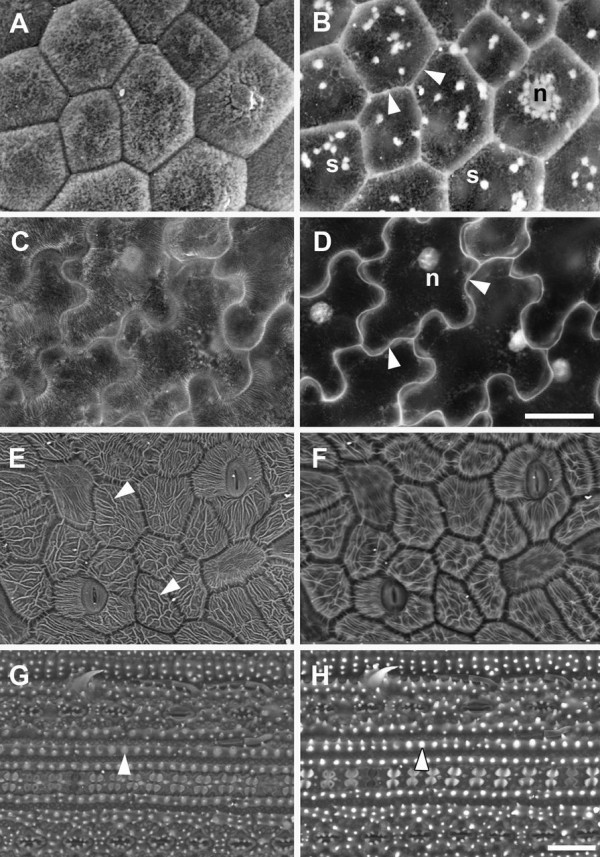
**BSE imaging of epidermal cell surface features and internal organelles in a critical point dried *A. thaliana *seed (A,B), cotton flower petal (C,D), cotton leaf (E,F) and rice leaf (G, H).** The same areas of tissue were imaged using 10 kV **(A**,**C**,**E**,**G)** or 20 kV **(B**, **D**, **F**, **H)** accelerating voltages (10 Pa chamber pressure). Arrowheads in **(B)** and **(D)** indicate cell outlines, in **(E)** indicate wax deposits on leaf surface, and in **(G)** and **(H)** silica deposits on the leaf surface. n = nucleus; s = starch granules. Scale bars = 20 μm (A–D; bar shown in D), 30 μm (**E**–**H**; bar shown in H).

Surface features, wall outlines and internal organelles can be seen in BSE images, depending on accelerating voltage. We extended our analysis to investigate the ability of the BSE detector to resolve these features in a variety of other species and tissues (Figure 7). In both developing seed of *A. thaliana* and cotton flower petals epidermal surface details were detected at 10 kV accelerating voltage (Figure 7A,C), while at 20 kV cell outlines and some internal organelles were seen (Figure 7B,D). In other tissues, surface deposits compromised cell outline contrast even at accelerating voltages of 20 kV or higher, as illustrated in cotton and rice leaves (Figure 7E-H). Cotton leaves contained distinct ridges of surface wax which contributed to the BSE signal (Figure 7E), and increasing the accelerating voltage to 20 kV (Figure 7F) or 30 kV (not shown) did not increase cell wall contrast or greatly reduce signal from the surface waxes. In comparison, the wax deposits on cotton flower petals became 'transparent’ to the beam at 20 kV (Figure 7D). Rice leaves are covered with deposits of silica, which contributed to the high BSE signal, and cell outlines were obscured at 10 kV, 20 kV (Figure 7G,H respectively) and 30 kV (not shown).

**Figure 8:**
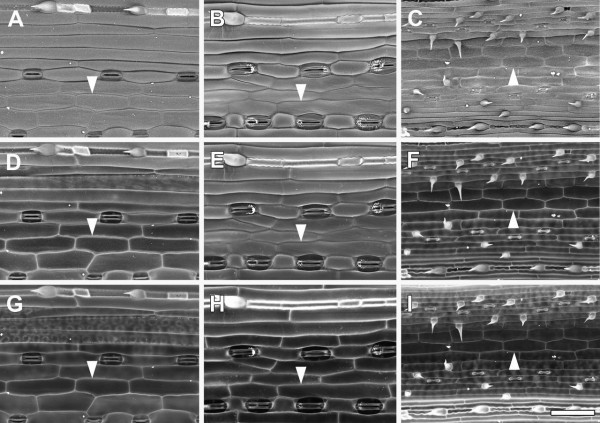
**Effect of accelerating voltage on BSE imaging of cell wall outlines in critical point dried barley (A,D,G), wheat (B,E,H) and *Brachypodium distachyon *(C,F,I) leaves.** The same leaf areas were imaged at 10 **(A**,**B**,**C)**, 20 **(D**, **E**, **F)** and 30 kV **(G**, **H**, **I)**, at 10 Pa chamber pressure. Arrowheads indicate cell wall outlines. Scale bar = 60 μm (A–I; bar shown in I).

We are often interested in quantifying cell size and shape in cereal tissues, including the model cereal *Brachypodium distachyon*. Barley leaf epidermal cells were analysed in this way by [17], as shown here (Figure 8A,D,G). BSE imaging at 10 kV revealed the epidermal surface in barley (Figure 8A), wheat (Figure 8B) and *Brachypodium* (Figure 8C) leaves. Increasing contrast was evident with higher accelerating voltages, and at 20 kV cell outlines were evident in barley and *Brachypodium* leaves (Figure 8 D,F respectively) but 30 kV was required for wheat leaves (Figure 8H).

### Origin of bright cell wall outlines

**Figure 9:**
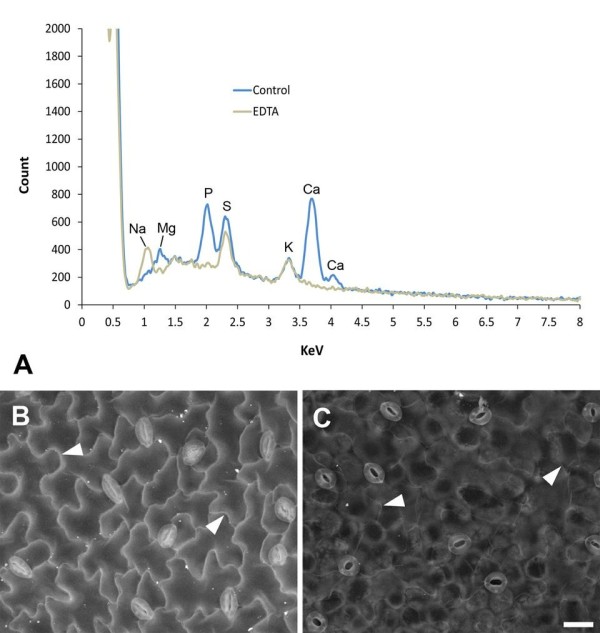
**X-ray microanalysis of critical point dried *A. thaliana *leaves (carbon-coated) and effect of EDTA chelation on BSE signal. (A)** EDS spectra from untreated leaves fixed in methanol ('control’) or leaves extracted with 1% EDTA overnight and fixed in methanol ('EDTA’). Note the lack of Mg and Ca peaks in EDTA-extracted leaves (both cations are extracted) and appearance of Na peak (most likely due to the Na present in the EDTA salt solution) in EDTA treated tissue. EDS spectra were collected from a 590x440 μm field (similar to images shown in B and C) at 20 mm working distance using 20 kV accelerating voltage and a spot size of 550 (1.7 nA probe current). Spectra were scaled to exclude lower atomic number elements including carbon (originating from the carbon coating). **(B**-**C)** BSE signal from cell wall junctions is relatively bright in untreated tissue (B) and weak in EDTA-extracted tissue **(C)**; arrowheads indicate cell wall outlines. Scale bar = 20 μm (**B,C**; bar shown in **C**).

Since there is a relationship between average atomic number and BSE signal [14], the brighter signal from cell wall outlines likely originates from higher atomic number components within the wall. To ascertain the possible contribution of different elements to the BSE signal, EDS spectra were acquired from critical point dried *A. thaliana* (Figure 9) and barley leaves (Additional file 2) at the same accelerating voltage used to obtain BSE images of wall outlines (20 kV). Since SEM processing results in leaching and relocation of elements nothing can be inferred from these analyses about concentration or original distributions of elements.

In *A. thaliana* leaves, the strongest peaks detected were from calcium, phosphorus and sulphur with smaller contributions from magnesium and potassium (Figure 9A), all of which are normally present in plant tissues [21,22]. Since there is generally a significant proportion of calcium in cell walls, we tested its contribution to the BSE signal by chelation of bound calcium with EDTA; this treatment resulted in a loss of BSE signal from epidermal cell walls (Figure 9C cf. Figure 9B), and loss of calcium peaks from the EDS spectra (Figure 9A). Note that EDTA treatment also resulted in loss of the magnesium peak (Figure 9A), since EDTA chelates both cations [23]. In comparison, EDS analysis of barley leaves suggested that there is a strong contribution from potassium in this tissue (Additional file 2). Collection of x-ray maps (to correlate BSE signal with element distributions) was not informative, since resolution is low and very long acquisition times (> 2 h) are required, which results in specimen damage.

### Image analysis

**Figure 10:**
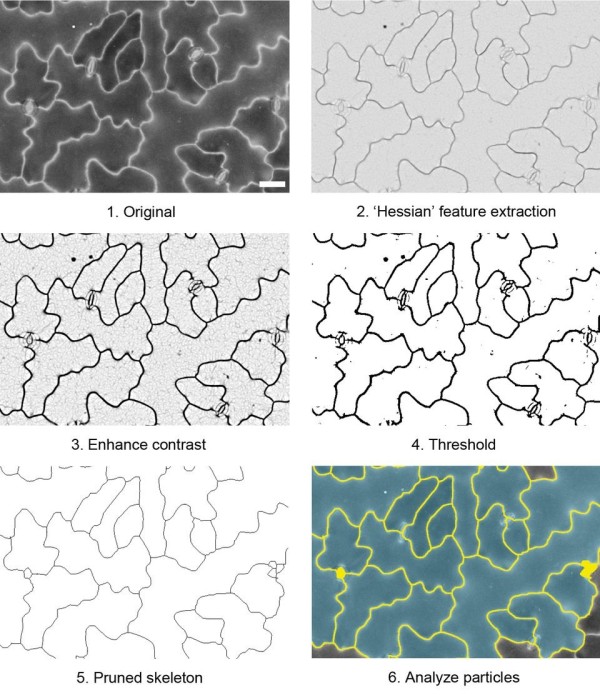
**Processing of BSE images of *A. thaliana *leaf for analysis of cell size. A summary of the main steps in the procedure are shown here for an example image (provided as Additional file****4****).** Not all the steps are shown here; a full description of the procedure and a macro are provided in Additional file 3. Scale bar = 30 μm in original image (1). Colours in step 6 ('Analyze particles’) indicate masks defining cell wall outlines (yellow) and cell areas (blue) in final processed image.

BSE images of epidermal cell wall outlines in *A. thaliana* leaves are ideal for image analysis as they contain high contrast information which lends itself to semi-automated cell size analysis (e.g. Figure 10). We used the image processing program Fiji, a distribution of the popular open source program ImageJ [24,25], to develop a simple procedure to analyze cell size from thresholded BSE images; this procedure is outlined in Figure 10 and in more detail in Additional file 3. First, the plugin 'FeatureJ Hessian’ [26] was implemented to discern edges (cell boundaries), and contrast was enhanced. A threshold was applied to the resultant image, which was then skeletonized, pruned and dilated to a standard amount to approximate cell boundaries. The 'Analyze Particles’ command was then used to measure cell area and other parameters. The procedure was recorded as a macro (provided in Additional file 3), which can be copied and pasted into the macro editor in the program. This macro was created by testing the processing and analysis steps in Fiji on the image in Additional file 4, a BSE image of *A. thaliana* leaf epidermis taken at 20 kV (similar to Figure 2C). The macro was then tested on other cell types (e.g., barley leaf, Additional file 5).

The magnification selected to capture images for analysis depends on how many cells can be accurately outlined and measured by the software. In this case images of *A. thaliana* leaves were taken at 200× magnification at a resolution of 1024 × 768 pixels, from which 30–40 cells were measured. Larger areas may be analysed if images from adjacent areas are stitched together beforehand, or the image is captured at higher resolution; we recommend testing several magnifications at different image resolutions to determine optimal image capture settings.

It must be noted that stomatal guard cells were lost from these images during processing. If stomatal cells are to be included in the analysis, a more detailed processing procedure should be developed, since guard cell walls are much less bright than surrounding pavement epidermal cell boundaries (Additional file 6). For analysis it is also important to avoid wrinkles in the walls (as a result of tissue shrinkage) as these will add to the signal and produce artefactual cell 'boundaries’ when processing the image for analysis. Critical point drying directly after methanol fixation and transfer to ethanol minimises such artefacts [20].

### Comparison with other methods to highlight cell outlines

**Figure 11:**
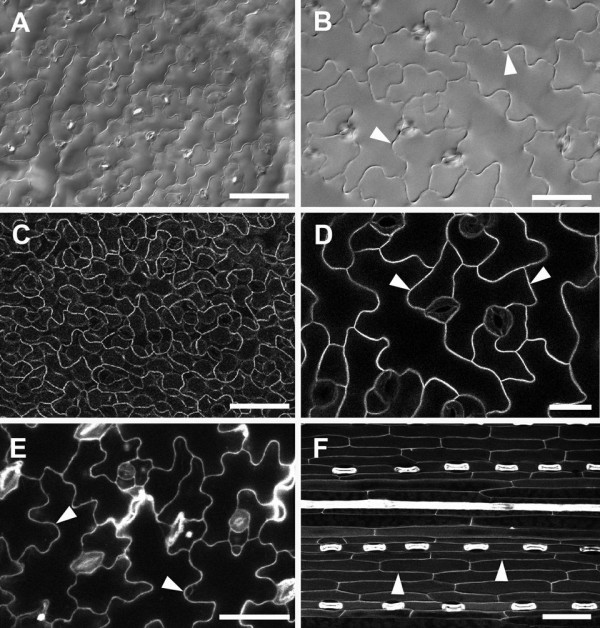
**Imaging of epidermal cells with widefield and confocal microscopy.** Cleared *A. thaliana* rosette leaves imaged by differential interference contrast (DIC) widefield microscopy **(A**, **B)**. Fresh *A. thaliana* leaves imaged by CLSM using GFP targeted to the plasma membrane **(C**, **D)**. CLSM imaging of propidium iodide stained fresh *A. thaliana* cotyledon **(E)**. Autofluorescence of a fresh barley leaf detected after UV excitation in CLSM **(F)**. Arrowheads indicate cell wall outlines. Scale bars = 30 μm (D), 50 μm **(B**, **E)**, and 100 μm **(A**, **C**, **F)**.

Differential interference contrast (DIC) is a relatively quick and easy method for obtaining light microscope images of cell outlines. When tissue was cleared using saturated chloral hydrate, the epidermal cells of *A. thaliana* could be seen using DIC on a compound microscope (Figure 11A). However, the images were usually low contrast, and out-of-focus blur from underlying tissues could also be seen (Figure 11A,B), which makes it difficult to use such an image for analysis. Many analysis packages also have difficulty identifying cell outlines when they are of varying grey levels, although this is improving with more sophisticated algorithms.

*A. thaliana* plants expressing GFP targeted to the cell surface (Figure 11C and D) and staining with cell wall binding dyes, such as propidium iodide (Figure 11E) can also be used to detect cell outlines. However, *A. thaliana* cotyledons and leaves are rarely flat and a Z-series must be collected to overcome this topography, and it is then difficult to distinguish between cell types in a maximum projection image derived from a Z stack showing cell surface GFP (Figure 11C). The cell walls of young *A. thaliana* cotyledons appeared to stain readily with propidium iodide (Figure 11E), but true leaves stained only after initial vacuum treatment (Additional file 7; [5]). Since the dye did not easily penetrate below the epidermis, this has the advantage of avoiding confusion between cell layers. Nevertheless, although contrast was high in individual sections of a Z-stack (Figure 11E; Additional file 7A), fluorescence from the periclinal wall of the epidermis reduced contrast of the anticlinal walls in maximum Z-stack projections (e.g., Additional file 7B).

Cell wall autofluorescence can also be used to obtain cell outlines. While *A. thaliana* leaf tissue exhibits little cell wall autofluorescence, cereal leaves contain brightly autofluorescent wall components [27]. Outlines are readily detected under UV excitation, but as above, a Z-series or image stitching may be required to overcome tissue topography. Since the epidermis is more autofluorescent than the underlying cells however, a relatively clear image of epidermal cell outlines was obtained (Figure 11F).

## Discussion

Variable pressure (VP)-SEMs allow detection of signals under low vacuum, enabling the use of minimally-processed, uncoated tissues. This study showed that imaging uncoated samples allowed detection of cell outlines with the BSE detector, information which is difficult to obtain using VP-SE or conventional high vacuum SE detectors. The technique outlined here also overcomes problems encountered when attempting to resolve cell outlines using other imaging methods.

### Cell wall outline imaging with the BSE detector

High contrast detection of cell outlines is a specific feature of plant cell walls observed via backscattered electrons in the SEM. Good resolution sufficient for straightforward image processing and analysis was obtained due to the generally bright BSE signal arising from epidermal cell walls. Using the BSE detector cell outlines in uncoated tissues were easily resolved under a wide range of imaging conditions in both VP and high vacuum modes, since backscattered electrons are relatively insensitive to charging artefacts. Although the VP-SE detector produces usable images in some cases, in most tissues high contrast cell boundaries were only observed with the BSE detector. Shown below is a summary of the recommended method and conditions used to obtain high contrast images suitable for analysis of cell size and shape.

1. Fix tissue in methanol for 10 min or longer. Vacuum infiltrate if necessary.

2. Dehydrate further in dry ethanol for 1 h (small tissues) or overnight (large tissues).

3. Critical point dry following manufacturer’s recommendations.

4. Mount tissue on SEM stub and observe as soon as possible (same or next day).

Recommended microscope operating conditions:

••Accelerating voltage = 20 kV (10 kV for surface topography)

••Chamber pressure = 10–40 Pa

••Working distance = 7 mm (check optimum distance for your detector)

••Spot size = 0.7 nA

••Increase image contrast to enhance cell wall outline contrast

5. If charging is a problem, ensure good contact of tissue with the stub and apply carbon paste to the edges of the tissue. If charging remains, coat tissue with carbon.

6. Store tissue in a desiccator or low humidity cabinet.

### Origin of cell wall outline contrast with the BSE detector

Backscattered electrons contribute to both of the contrast mechanisms underlying image formation in the SEM; compositional (atomic number) contrast and topographic contrast [14]. Compositional contrast most likely explains the bright signal from cell walls in most tissues observed in this study. Plant cell walls contain varying amounts of calcium, phosphorous, silicon, sulphur, potassium, magnesium and chloride, depending on the species and tissue [21,22]. These components produce higher BSE yields compared to lower atomic number organic constituents (carbon, hydrogen and oxygen) in the cell wall and cytoplasm [28]. Some cell walls normally accumulate ions, for example, trichome walls (e.g., *A. thaliana*; [29]), which increases endogenous BSE contrast (e.g., Figure 1B). X-ray microanalysis data suggested that calcium in *A. thaliana* (Figure 9) and potassium in barley (Additional file 2) leaf tissue were the main constituents underlying the strong BSE signals in epidermal cell walls.

Calcium is a likely candidate as a source of BSE signal at cell junctions. Plant cell walls preferentially accumulate cations, since carboxyl groups on demethylated pectin, and to a lesser extent on cellulose and proteins, impart an overall negative charge [30,31]. Calcium is normally bound to demethylated pectin in walls, and is enriched at cell junctions, which strengthens cell-cell adhesion [32]. Interestingly, it has recently been shown that propidium iodide competes with calcium in binding to carboxyl groups on demethylated pectin [7], explaining why cell wall outlines revealed by propidium iodide (Figure 11E and Additional file 7) are very similar to those observed with the BSE detector (e.g., Figure 2C). Specimen processing results in leaching and relocation of un-bound ions from cells [33] and it is likely that cations not originally located in the wall, including calcium, magnesium and potassium, accumulate at unoccupied anionic sites within the wall during preparation of tissues for SEM. In this way, removal of water may create additional compositional contrast at wall boundaries for BSE imaging.

Although plant tissues are optimally preserved for SEM in the frozen state [34], and EP-SEM is beneficial for imaging certain tissues [16], no bright wall outlines could be seen in frozen tissues or in fresh tissues observed with EP-SEM (Figure 6). Epidermal cell outlines were visible, but these were generated by topography of epidermal cells, and were of low contrast compared to outlines observed in critical point-dried tissue (e.g., Figure 2C). Furthermore, there are disadvantages to using either low temperature VP-SEM or EP-SEM imaging to obtain images of cell outlines. A common drawback is that imaging with either method needs to be quick, as the tissue will freeze-dry due to sublimation during imaging of frozen tissue (without a dedicated liquid nitrogen-cooled cryo-SEM stage). Tissue also loses water rapidly when imaging with EP-SEM, and tissues viewed by either method are sensitive to beam damage. A final disadvantage is that the tissue cannot be stored and re-imaged if required. Nevertheless, imaging frozen tissue either with [35] or without [36] a Peltier-cooled stage may be of use to quickly examine uncoated tissues in the VP-SEM without the need for the dedicated and expensive cryo-preparation equipment required for longer analysis of tissue held at close to liquid nitrogen temperatures.

### Comparison with other techniques for visualizing cell outlines

CLSM images yield high-contrast cell outlines only when sufficient fluorescence can be obtained from cell walls or membranes, either from staining or localised GFP expression. The waxy cuticles found on most plant epidermal surfaces are generally quite hydrophobic, and therefore commonly used aqueous stains, including propidium iodide, may not penetrate to stain periclinal cell walls without vacuum infiltration or pre-treatment to remove some surface wax. However, such treatments must not be so harsh that cell membrane integrity is compromised, since for high contrast, cell wall stains must be retained in the apoplastic space, and excluded from the cytoplasm.

Topography is often a problem because confocal images are generated from a thin optical slice of tissue, and information from a complete 3D surface can generally be obtained only from a z-stack. However, isolating epidermal fluorescence from such stacks is time-consuming. Topography may be overcome by stitching adjacent images from slightly different focal planes, but this is also time-consuming and may require manual checking even if image capture (with autofocus) and draft stitching can be automated.

Many plant tissues show cell wall autofluorescence, and high contrast cell outlines can be obtained from many cereal tissues, which generally show strong blue-green fluorescence with UV excitation [27]. However, even if present, cell wall autofluorescence may be insufficient for the high contrast outlines required for image analysis, and BSE images provide superior contrast.

### Caveats

One potential problem with SEM observation, particularly in relation to image quantification, is that processing tissue through fixation, dehydration and critical point drying (CPD) can lead to tissue shrinkage [18,19], and changes in cell size. We have found that concomitant fixation and dehydration in 100% methanol followed by transfer to ethanol prior to CPD resulted in the least tissue shrinkage and best preservation of tissue morphology [20]. An advantage of this fixation method is that it is very quick; many tissues can be processed for imaging within 2–3 h. Original tissue dimensions will be preserved as faithfully as possible if the tissue is viewed soon after processing, and stored in a desiccated or low-humidity environment for future imaging if necessary.

As noted earlier, most epidermal surfaces have surface elaborations such as waxy cuticles or mineral deposits. In some cases, the primary electron beam can penetrate these coverings (e.g. Figure 7D), but heavily elaborated tissues cannot be analysed this way (Figure 7F,H). Furthermore, additional SE or BSE signal from organelles and other cytoplasmic structures (Figure 7B,D; [15]) may interfere with the ability to capture clear cell wall outlines. Another limitation is that only relatively flat tissue is suitable for imaging in order that cell size is faithfully represented. However, it is possible to orient tissue on the SEM stub or rotate the stage in order to image the epidermal area of interest. As with all preparation techniques it is advisable to first assess the suitability of this method for the cells or tissues of interest.

### Image analysis

The technique presented here for obtaining high contrast images suitable for analysis of epidermal cell size and shape is relatively quick and simple, and with the rising popularity of affordable desktop SEMs, this protocol provides a good alternative to other imaging methods. Fortuitously, BSE imaging of cell outlines is well-suited to epidermal cells since they generally contain large vacuoles and have little cytoplasm with few organelles. There are very many image analysis packages and protocols available for processing images; the processing and analysis steps shown here for ImageJ/Fiji can be readily adapted to an institution’s preferred analysis package.

## Conclusions

For many plant tissues, quantification of cell surface size and shape can be done rapidly using the protocol outlined above with relatively few artefacts. Imaging uncoated tissue in the variable-pressure SEM using the BSE detector is straightforward and provides a simple protocol for laboratories with standard SEM processing equipment. Furthermore, tissues can be processed in batch, examined and stored for future imaging if required.

## Methods

### Plant material

Tissues from a number of different plants were prepared for SEM, including *Arabidopsis thaliana* (L.) Heynh (Columbia), *Brachypodium distachyon* L. (21–3 line), *Gossypium barbadense* L. (cotton; Pima variety), *Gossypium hirsutum* L. (cotton; Coker variety), *Hordeum vulgare* L. (barley; Himalaya cultivar), *Oryza sativa* L. (rice; Nipponbare), and *Triticum aestivum* L. (wheat; Bobwhite). Tissues were dissected from the plant and immediately fixed in 70% ethanol for a minimum of 30 min, or 100% anhydrous methanol for 10 min [20,37], at room temperature. Application of light vacuum within the first 5 min (or until tissue sank) improved ethanol fixation of more difficult tissues (e.g., rice leaves), although tissue sank much faster in methanol even without vacuum treatment. For ethanol fixation, tissues were dehydrated to 100% anhydrous ethanol in 10% steps, 30 min each step (100% ethanol was changed twice, 30 min each). For methanol fixation, methanol was replaced with 100% anhydrous ethanol twice, 30 min each; larger tissue pieces were left overnight in ethanol [20]. After a rinse in 100% anhydrous ethanol, tissues were critical point dried with an Autosamdri-815 automatic critical point drier (Tousimis Research Corporation, Rockville USA). Artefacts, such as shrinkage of tissue and cell wall wrinkling, were minimized by methanol fixation [20,37] and by processing tissue straight through to critical point drying within 1–2 hours of reaching the second 100% ethanol change, or the day after fixation and dehydration at the latest. Methanol fixation is recommended as we generally found it to be superior to ethanol and other commonly used SEM preparation procedures [20].

### Variable Pressure-SEM

Specimens were mounted on aluminium stubs with double-sided sticky carbon tabs (ProSciTech, Qld, Australia) and visualized uncoated in a Zeiss EVO LS 15 Extended Pressure-Scanning Electron Microscope (Carl Zeiss Pty Ltd, Sydney, Australia) in variable-pressure (VP) mode (with nitrogen as the imaging gas), with a final VP aperture of 100 μm. The backscattered electron detector was a 4-quadrant solid state type mounted below the final aperture directly above the sample. Other instrument settings are detailed in the text and figure captions. If charging was excessive between 10 and 50 Pa, tissue was removed from the chamber and carbon paste was applied to the edges, improving contact between sample and stub. If charging was still present at up to 100 Pa the tissue was coated with carbon (~30-40 nm) using an Emitech K500X sputter coater with K250 carbon coating attachment (Quorum Emitech, Kent, UK). For comparison, some tissue pieces were sputter coated with gold (~20 nm). To reduce absorption of moisture or further changes in tissue dimensions, critical point dried tissue was stored (mounted or un-mounted) in a electronic humidity-controlled storage cabinet set at 10% relative humidity (Thermoline Scientific, Australia).

### Low temperature VP-SEM and extended-pressure-SEM (EP-SEM)

For low-temperature VP-SEM, leaves were dissected from the plant and immediately mounted on a drop of water on a 9 mm stub with a double-sided sticky carbon tab. The stub and tissue were frozen in liquid nitrogen then transferred to the Deben Coolstage which had been pre-cooled to -20-30°C [35]. This procedure provides approximately 20 min imaging time before the tissue freeze-dries in the vacuum due to sublimation of water. A dedicated cryo-stage which enables imaging of tissue at liquid nitrogen temperatures is best, but such dedicated equipment is not easily accessible or affordable for most laboratories.

For extended pressure SEM (EP-SEM), leaves from 14-day old agar-grown *A. thaliana* Columbia seedlings were dissected and immediately mounted on a 9 mm stub with a double-sided sticky carbon tab. Several small drops of distilled water were placed around the tissue to maintain local humidity, and the stub was transferred to a Deben Coolstage (Peltier-cooled stage; Deben, UK) attached to the Zeiss EVO LS15 and the chamber pumped down. To maintain tissue in a hydrated state, EP-SEM conditions were 82% humidity, 600–700 Pa chamber pressure, 2-3°C Peltier stage temperature, and 20–25 kV accelerating voltage.

### EDS microanalysis

For X-ray microanalysis, critical point dried 14-day old agar-grown *A. thaliana* Columbia leaves and barley leaf pieces were analyzed with an Oxford Inca PentaFetx3 SiLi detector with a 30 mm^2^ ATW2 window (Oxford Instruments). Leaves were carbon coated to avoid excessive charging (see above) and analyzed under high vacuum using 20 kV accelerating voltage (1.7 nA probe current; 150 μA beam current) at 18-20 mm working distance and approximately 400× magnification. Spectra were acquired over 2 min; peaks were manually confirmed in the software (INCA suite v. 4.11). Background spectra from different areas of the stub (carbon tab) were acquired under the same conditions for comparison. To test the contribution of calcium to the BSE signal, freshly harvested *A. thaliana* leaves were extracted with 1% ethyelenediaminetetracetic acid (EDTA, sodium salt). Leaves were first vacuum infiltrated with the solution and left overnight. Pieces were washed in water, fixed in methanol and dehydrated in ethanol (see above), and critical point dried. Control leaves were fixed in methanol, dehydrated in ethanol and critical point dried.

### Light and Confocal microscopy

For Differential Interference Contrast (DIC) imaging, leaves from 14-day old agar-grown *A. thaliana* Columbia seedlings were cleared in saturated chloral hydrate overnight. Cleared leaves were mounted in 50% glycerol and observed with a Zeiss Axioimager M1 compound microscope using DIC optics. For fluorescence visualization of cell outlines in *A. thaliana*, cotyledons from 7-day old agar-grown Columbia seedlings were dissected from the plant and mounted directly in 10 μg/ml propidium iodide. After 10 min they were observed on a Leica TCS SP2 CLSM using 488 nm excitation and 560–620 nm emission. Similarly, *A. thaliana* leaves from 3 week old seedlings were cut at the petiole and infiltrated with 100 μg/ml propidium iodide under light vacuum [5], for 3 × 1 min. For visualization of GFP in *A. thaliana* line 29–1 (plasma-membrane localized GFP; [10]), leaves were dissected from 3–4 week old agar-grown or soil-grown seedlings, mounted in water and observed using 488 nm excitation and 500–530 nm emission. Barley leaf pieces were dissected and mounted in silicone oil, and autofluorescence between 420–580 nm was detected following UV (405 nm) excitation.

## Competing interests

The authors declare that they have no competing interests.

## Authors’ contributions

MT contributed to conception and design of the study, carried out all experiments and analysis and drafted the manuscript. RW contributed to conception and design of the study and drafted the manuscript. Both authors read and approved the final manuscript.

## Supplementary Material

Additional file 1 **Calibration of the BSE detector.** Mean pixel grey values for images of a silicon chip wafer captured with a 4-quadrant solid-state BSE detector on a Zeiss EVO LS15 EP-SEM. Silicon was chosen as it gives a homogenous flat image from which an average pixel grey value can be calculated. Images were captured at decreasing working distances from 20 to 2 mm; brightness (50%) and contrast (35%) levels were unchanged. 20 kV accelerating voltage at 10 Pa chamber pressure and a spot size of 550 (1.7 nA probe current) was used. Click here for file

Additional file 2 **X-ray microanalysis of critical point dried barley leaves (carbon-coated).** EDS spectrum was acquired under the same conditions as those for *A. thaliana* leaves (Figure 9). As for spectra shown in Figure 9, the spectrum was scaled to exclude lower atomic number elements including carbon (originating from the carbon coating). Click here for file

Additional file 3 Workflow for semi-automated cell size analysis using Fiji (ImageJ version 1.47 h). Click here for file

Additional file 4 **BSE image of critical point dried *A. thaliana *leaf**. This image was used to test processing and analysis steps in Fiji (Additional file 3). Click here for file

Additional file 5 **BSE image of critical point dried barley leaf.** This image was processed using the Fiji image processing and analysis macro recorded for *A. thaliana* leaves (Additional files 3 and 4), without any modifications. (A) original and (B) processed image, with masks defining cell wall outlines (yellow) and cell areas (blue). Arrows indicate errors in recognizing some stomata and adjacent epidermal cells, most likely due to topographical contrast around the guard cells. Scale bar = 20 μm. Click here for file

Additional file 6 **BSE image of critical point dried *A. thaliana *leaf showing apparently disjunct boundaries between stomata (s) and pavement epidermal cells.** This is due to a small stretch of wall (arrowheads) close to the guard cells which has a much lower BSE signal than the adjoining pavement cell walls. Strong signal from the inner walls of guard cells (arrows), contrasts with weak signal from the outer walls (open arrowheads). These properties make recognition of stomata difficult during image processing. Scale bar = 20 μm. Click here for file

Additional file 7 **Propidium iodide staining of *A. thaliana *leaf epidermal cells.** The same field of epidermal cells showing an individual slice (A) and maximum projection (B) of a Z-stack. High contrast epidermal cell outlines (arrowheads) can be captured in an individual slice (A), but diffuse fluorescence of outer periclinal walls (stars) contribute to low contrast in the flattened stack (B). Arrows in (B) indicate intense fluorescence of inner guard cell walls of stomata (s). Scale bar = 20 μm. Click here for file
