# Supplementary figures and images for: Cell surface and cell outline imaging in plant tissues using the backscattered electron detector in a variable pressure scanning electron microscope
Source: Plant Methods. 2013 Oct 17;9:40. doi: 10.1186/1746-4811-9-40 (PMC3853341; doi:10.1186/1746-4811-9-40)

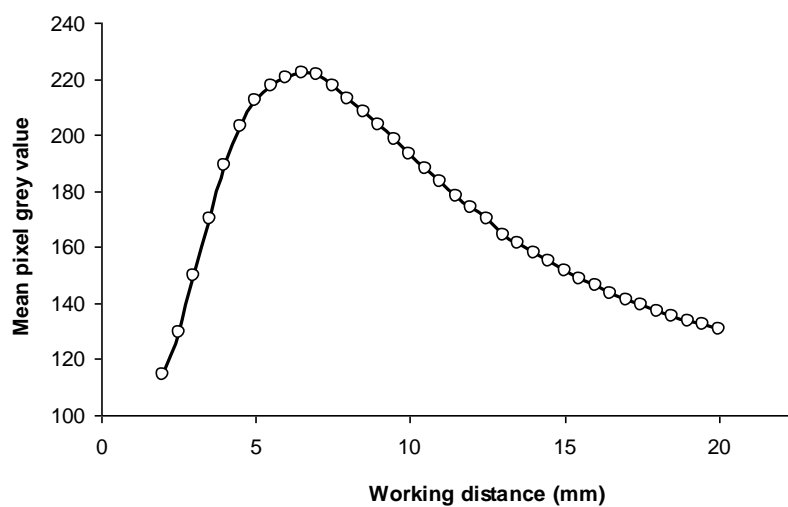

Supplement: Additional file 1 — Calibration of the BSE detector. Mean pixel grey values for images of a silicon chip wafer captured with a 4-quadrant solid-state BSE detector on a Zeiss EVO LS15 EP-SEM. Silicon was chosen as it gives a homogenous flat image from which an average pixel grey value can be calculated. Images were captured at decreasing working distances from 20 to 2 mm; brightness (50%) and contrast (35%) levels were unchanged. 20 kV accelerating voltage at 10 Pa chamber pressure and a spot size of 550 (1.7 nA probe current) was used. [file 1746-4811-9-40-S1.pdf]

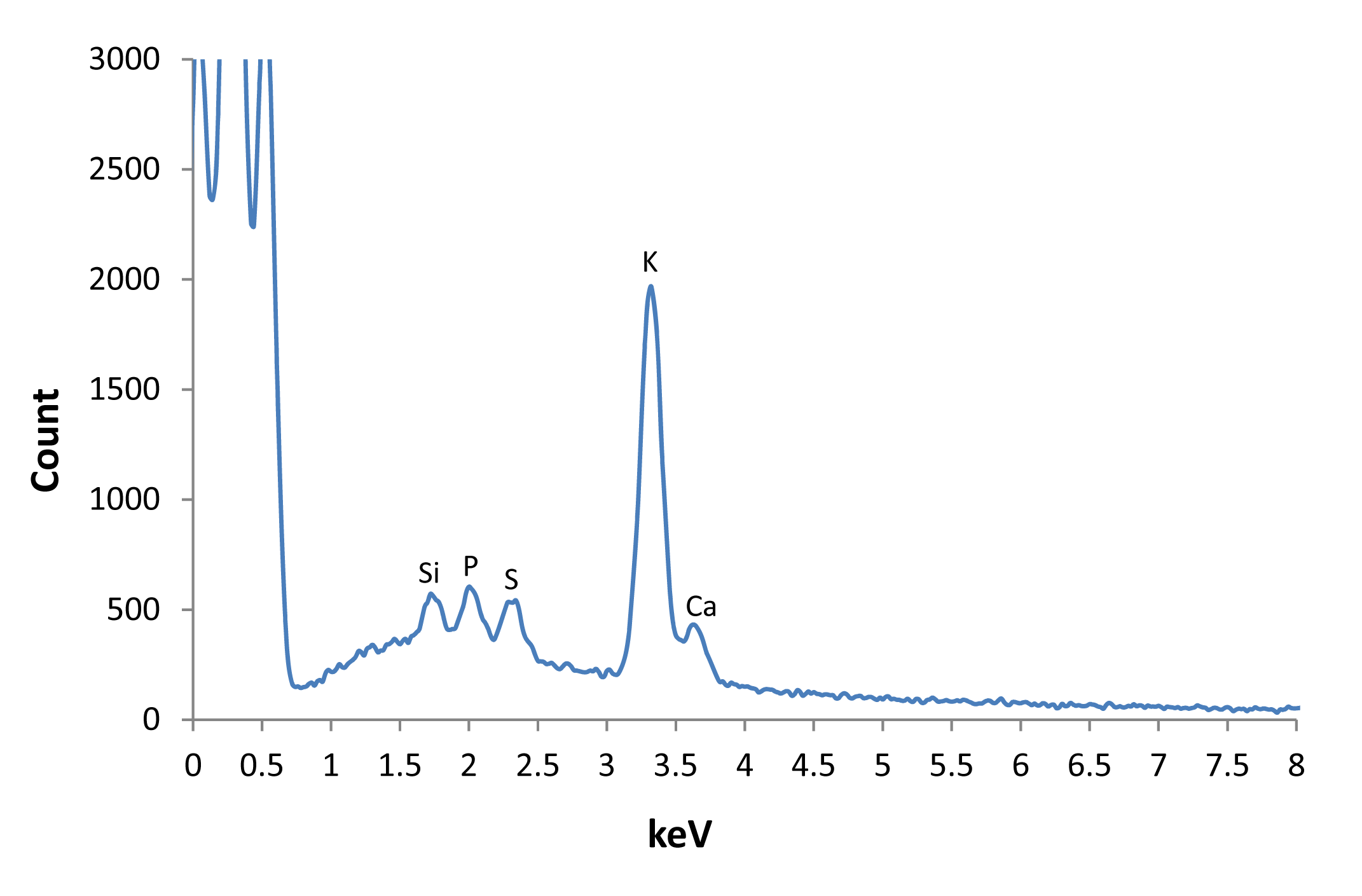

Supplement: Additional file 2 — X-ray microanalysis of critical point dried barley leaves (carbon-coated). EDS spectrum was acquired under the same conditions as those for A. thaliana leaves (Figure 9). As for spectra shown in Figure 9, the spectrum was scaled to exclude lower atomic number elements including carbon (originating from the carbon coating). [file 1746-4811-9-40-S2.tiff]

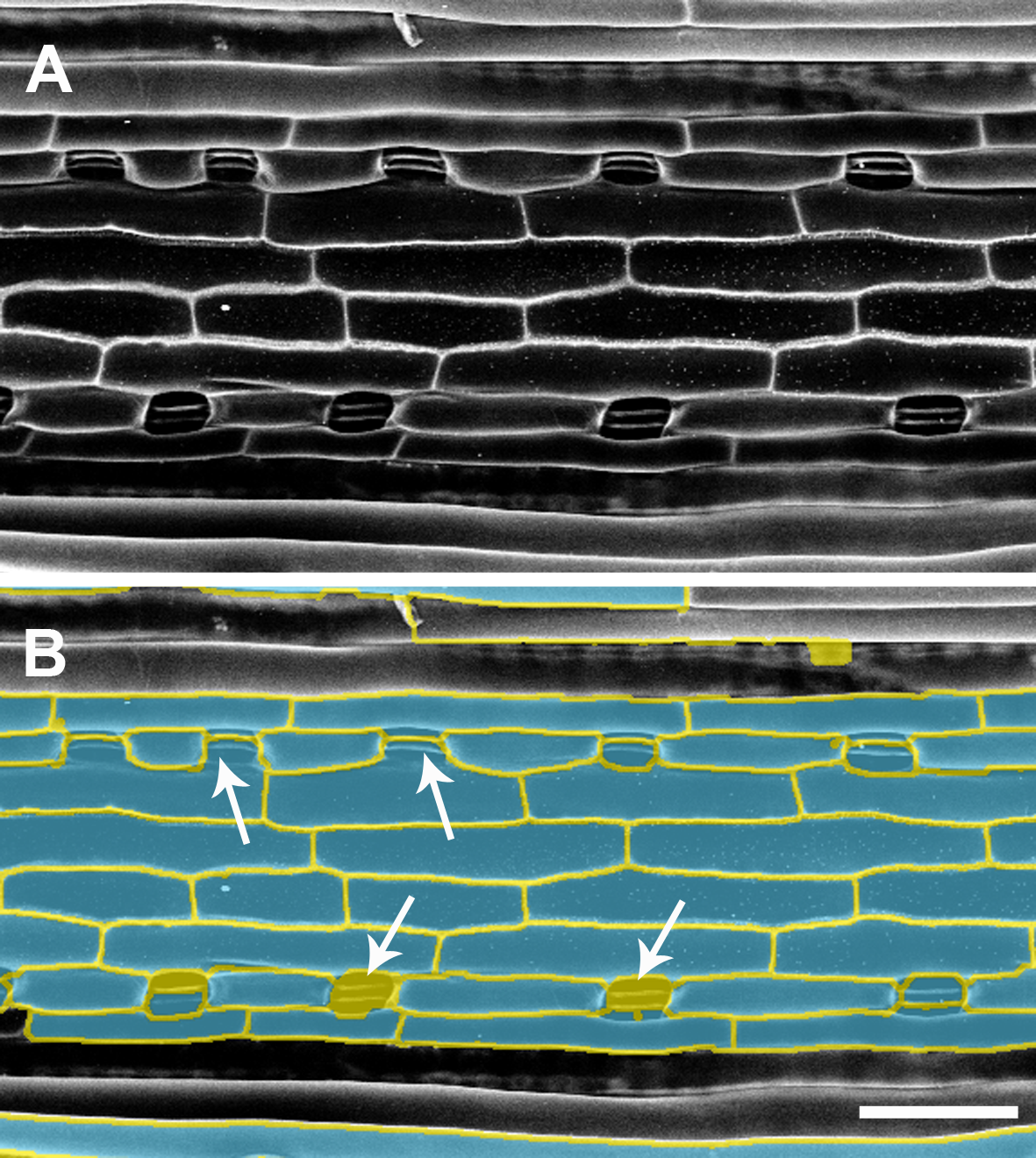

Supplement: Additional file 5 — BSE image of critical point dried barley leaf. This image was processed using the Fiji image processing and analysis macro recorded for A. thaliana leaves (Additional files 3 and 4), without any modifications. (A) original and (B) processed image, with masks defining cell wall outlines (yellow) and cell areas (blue). Arrows indicate errors in recognizing some stomata and adjacent epidermal cells, most likely due to topographical contrast around the guard cells. Scale bar = 20 μm. [file 1746-4811-9-40-S5.tiff]

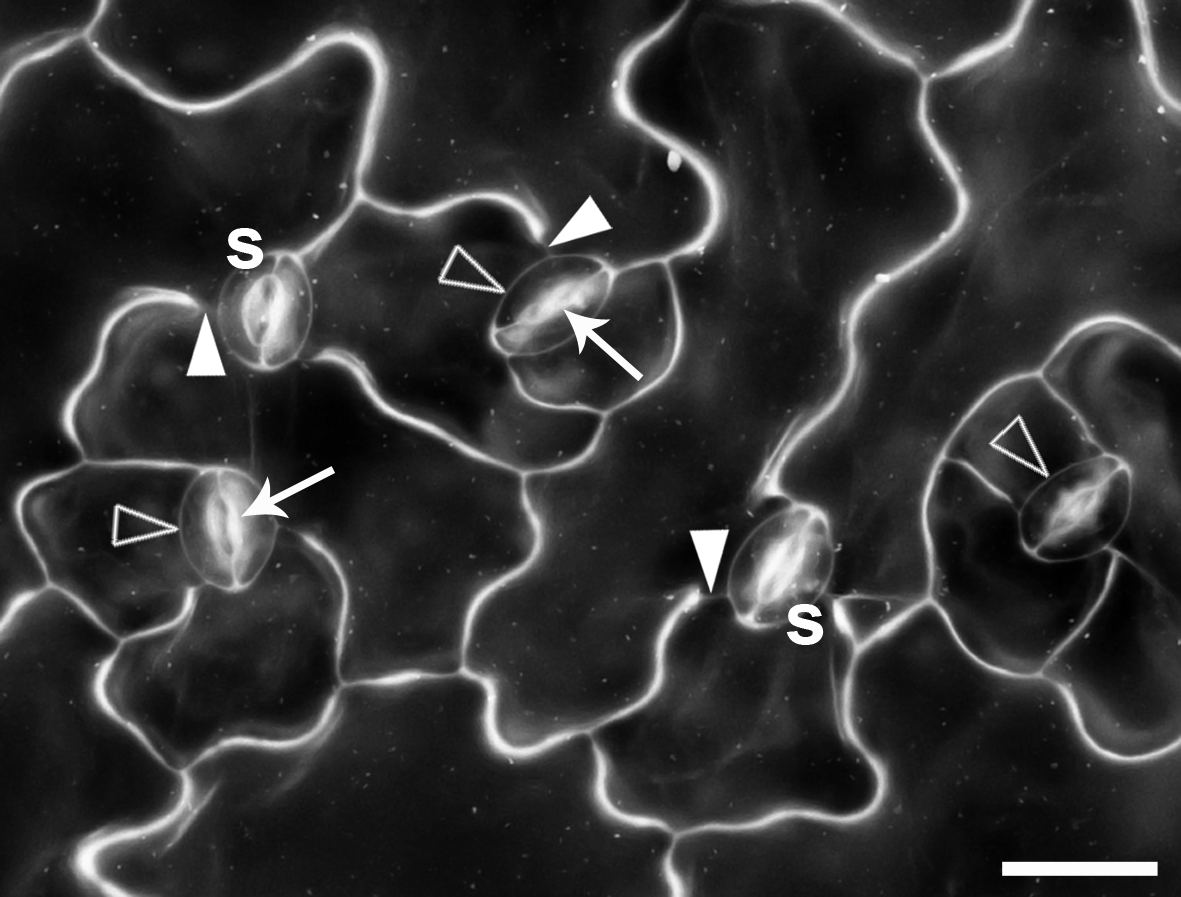

Supplement: Additional file 6 — BSE image of critical point dried A. thaliana leaf showing apparently disjunct boundaries between stomata (s) and pavement epidermal cells. This is due to a small stretch of wall (arrowheads) close to the guard cells which has a much lower BSE signal than the adjoining pavement cell walls. Strong signal from the inner walls of guard cells (arrows), contrasts with weak signal from the outer walls (open arrowheads). These properties make recognition of stomata difficult during image processing. Scale bar = 20 μm. [file 1746-4811-9-40-S6.tiff]

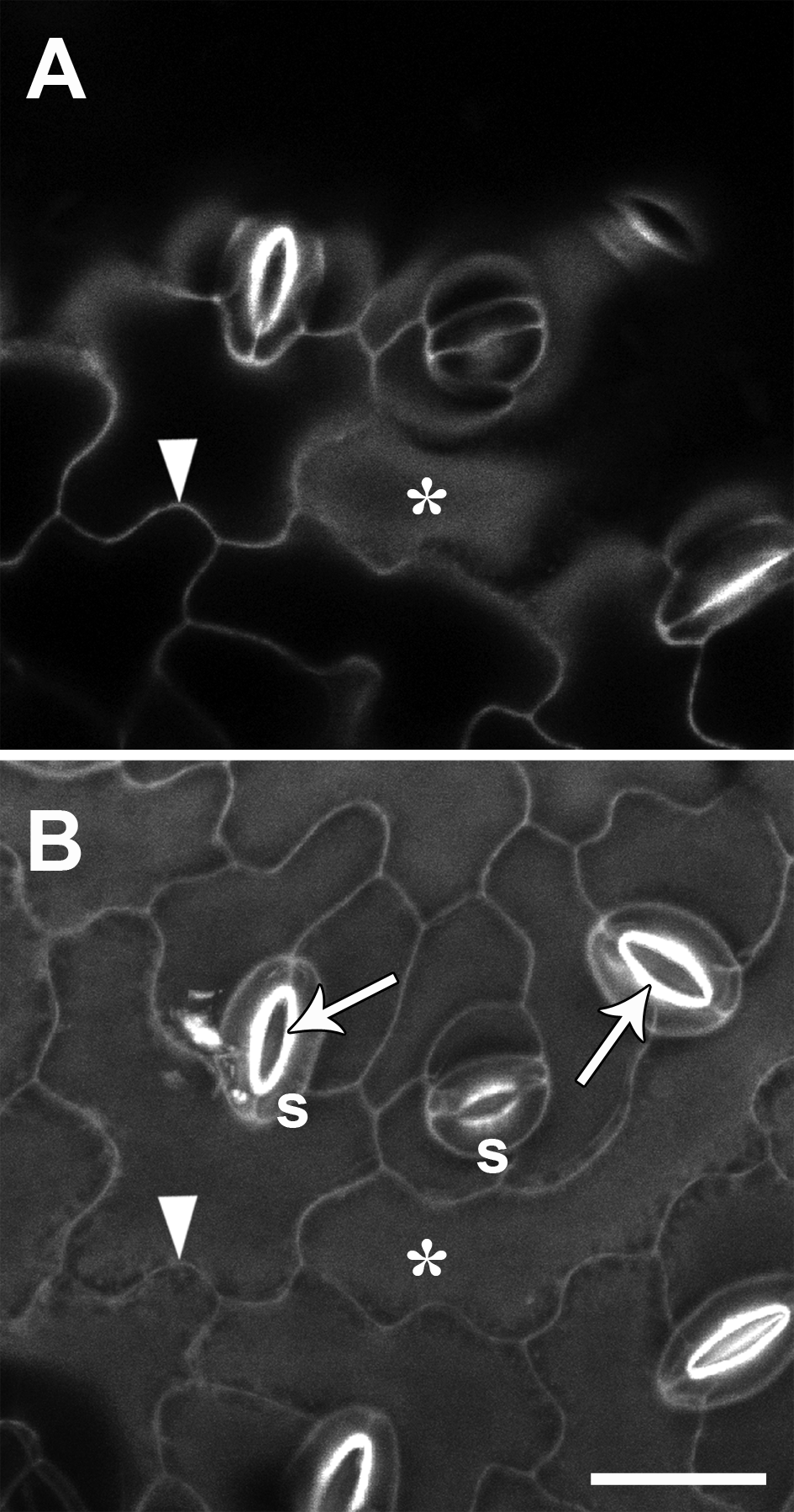

Supplement: Additional file 7 — Propidium iodide staining of A. thaliana leaf epidermal cells. The same field of epidermal cells showing an individual slice (A) and maximum projection (B) of a Z-stack. High contrast epidermal cell outlines (arrowheads) can be captured in an individual slice (A), but diffuse fluorescence of outer periclinal walls (stars) contribute to low contrast in the flattened stack (B). Arrows in (B) indicate intense fluorescence of inner guard cell walls of stomata (s). Scale bar = 20 μm. [file 1746-4811-9-40-S7.tiff]
