# Supplementary material for: Cell surface and cell outline imaging in plant tissues using the backscattered electron detector in a variable pressure scanning electron microscope
Source: Plant Methods. 2013 Oct 17;9:40. doi: 10.1186/1746-4811-9-40 (PMC3853341; doi:10.1186/1746-4811-9-40)
Supplement: Additional file 3 — Workflow for semi-automated cell size analysis using Fiji (ImageJ version 1.47 h). [file 1746-4811-9-40-S3.docx]

**Additional File 3**

**Workflow for semi-automated cell size analysis with Fiji (Image J version 1.47h)**

The instructions are written below with the assumption that all the images or set of images to be analyzed have the same magnification and capture resolution. Also, all procedures can be recorded in ImageJ/Fiji as a macro (see section 2 for a brief description of how to do this).

**Section 1. Processing and analysis**

*Preparing images – Setting scale*

1. Set the scale using scale bar, usually burnt into image by the SEM software. To do this, select the line tool on the toolbar and draw a straight line (hold down the ‘shift’ key when clicking and dragging to constrain in the horizontal direction) across the scale bar. Go to Analyze → Set scale. Type in the known distance (i.e., length of scale bar in μm).

*Note*: If all images have a scale bar, this needs to be cropped from all images before processing and analysis can proceed. If the images are all the same, a crop command can be recorded in the macro. Make a rectangular selection around the image excluding the scale bar, then go to Image → Crop.

If all images are taken at the same magnification then one image can be taken with the scale bar on as a scale ‘calibration’ image, and all others without (if this is possible in the SEM software), and the scale can be set ‘globally’ (click ‘Global’ in set scale dialog box) in ImgaeJ/Fiji so that all the images processed within a single session will have the same scale. (i.e., when the prorgam is closed this will resest).

*Processing images*

A macro was recorded for a ‘test’ image of *Arabidopsis thaliana* leaf (provided as Additional File 4), with the steps 1-10 outlined below. The macro is provided in Section 2 of these instructions. It is recommended to go through each of these steps sequentially with the image in Additional File 4, and several ‘test’ images, and modify the procedure if needed.

1. Convert image to 8-bit greyscale if necessary (Image → Type → 8-bit). 8-bit greyscale is needed for further processing.
2. Plugins → Feature extraction → FeatureJ → FeatureJ Hessian

Select “Smallest eigenvalue of Hessian tensor” only

Set “Smoothing scale” to 1.5

(For details of plugin, see:

<http://www.imagescience.org/meijering/software/featurej/hessian.html>)

1. Process → Enhance contrast

Saturated pixels = 10%

Select “Normalize” only

1. Process → Binary → Make binary

The image can also be thresholded manually by going to Image → Adjust → Threshold

1. Process → Binary → Options.

Iterations = 3

Count = 1

EDM output = overwrite

Do = Dilate

1. Process → Binary → Skeletonize
2. Plugins → Maros → Run → “PruneAll.txt”

Macro is available from the morphology collection by Gabriel Landini

<http://www.dentistry.bham.ac.uk/landinig/software/software.html>

Download entire Morphology set, and once extracted as per instructions on website, “PruneAll.txt” can be copied into the macros folder in ImageJ directory for easy access.

1. Process → Binary → Options.

Iterations = 1

Count = 1

EDM output = overwrite

Do = Dilate

1. Edit → Invert

*Analysis*

1. Analyze → Analyze Particles

Size: 200-infinity (Pixel units selected)

Circularity: 0.00-1.00

Show: outlines

Select “Display results”, “Clear results” and “Exlcude on edges”

**Section 2. Creating and running a macro**

*Create a macro*

Once all the processing and analysis steps have been tested or modified a macro can be recorded to automate analysis of images. Open a test image, then go to Plugins → Macros → Record; a ‘Recorder’ dialogue box will appear. From this posint on all steps are recorded as lines of text in this box. Note that between each step this text can be modified, or unwated steps deleted. To record, repeat every step shown above (or modified steps as determined for different images). At the end click ‘Create’ and the software will open a new macro window, from which the macro can be edited or saved. A sample macro is shown below, which was recorded for the image shown in Additional File 4 (note that this image still has the data bar on which will need to be cropped (see above)). This sample macro can be used as a test run and modified depending on the images being analyzed.

To create a macro from the sample below, copy and paste the text into a new macro window (Plugins → New → Macro) and save in the ‘macros’ folder within the Fiji directory.

run("8-bit");

run("FeatureJ Hessian", " smallest smoothing=1.5");

run("Enhance Contrast...", "saturated=10 normalize");

run("Make Binary");

run("Options...", "iterations=3 count=1 edm=Overwrite do=Dilate");

run("Skeletonize");

runMacro("PruneAll.txt");

run("Options...", "iterations=1 count=1 edm=Overwrite do=Dilate");

run("Invert");

run("Analyze Particles...", "size=200-Infinity pixel circularity=0.00-1.00 show=Outlines display exclude clear");

*Running macro on single images or all images in a directory*

This section describes how the macro can be run on single images or all images in a directory, but giving the user control for each image and collect results from each image separately. Macros can be run once by going to Plugins → Macros → Run. To run the macro multiple times on all images in a directory, Install the macro by going to Plugins → Macros → Install, which inserts the macro at the bottom of the ‘Plugins → Macros’ submenu; the macro will be accessible in this location for the entire session. Open an image, select the macro and it will run. To analyze the next image in the directory, click File → Open Next, and run the macro again. For each image, data from the results table can be copied and pasted into Excel or another program to analyze results. Note that images can be run in batch mode (Process → Batch → Macro) for automatic processing, but the results are not tabulated.
